# The Impact of Undergraduate Tutor System in Chinese 8-Year Medical Students in Scientific Research

**DOI:** 10.3389/fmed.2022.854132

**Published:** 2022-05-26

**Authors:** Yuxuan Liao, Hu Zhou, Fang Wang, Mingyi Zhao, Jianzhen Wu, Pengfei Rong

**Affiliations:** ^1^Department of Health Management, The Third Xiangya Hospital, Central South University, Changsha, China; ^2^Xiangya School of Medicine, Central South University, Changsha, China; ^3^Department of Endocrinology, The Third Xiangya Hospital of Central South University, Changsha, China; ^4^Department of Pediatric, The Third Xiangya Hospital of Central South University, Changsha, China; ^5^Department of Radiology, Third Xiangya Hospital of Central South University, Changsha, China

**Keywords:** Chinese 8-year medical students, undergraduate tutor system, undergraduate research, scientific research ability, questionnaire survey

## Abstract

**Purpose:**

This study aimed to evaluate the effect and influence of the undergraduate tutor system on the undergraduate stage of Chinese 8-year medical program students in scientific research.

**Methods:**

We collected related data from 194 medical students in the Xiangya Medical School of Central South University. The questionnaire was composed of three parts, namely, eight questions for basic information about individual and undergraduate tutor system, five questions for the subjective feeling impact of the undergraduate tutor system, and 22 questions for accessing the scientific research ability and academic results. The students were mainly divided into three groups to compare different kinds of undergraduate tutor systems, namely, single tutor for multiple students' system (group A), multiple tutors for multiple students' system (group B), and no tutor system for comparison (group C).

**Results:**

The type of tutorial system, the frequency of guidance, and the way of guidance were independent influence factors of the view of 8-year medical students on scientific research. Group B behaved better than group C in literature processing (*P* = 0.012), experimental operation (*P* < 0.001), statistical analysis (*P* < 0.001), and manuscript producing (*P* = 0.019). Group A and B joined in more National college students' innovation and entrepreneurship training programs (*P* = 0.003, *P* < 0.001). The most popular types of articles published by students were bioinformatics, meta-analysis, and reviews.

**Conclusion:**

Undergraduate tutor system has made tremendous achievements in cultivating students' scientific research capacity; however, implement improvement should be considered to better educate students.

## Introduction

As a Chinese characteristic medical education system, the 8-year medical program aims to cultivate distinguished medical doctor (M.D.) or philosophy doctor (Ph.D.). However, regardless of the type of doctors, the 8-year medical students are expected to be the leaders with innovative thinking and multidisciplinary crossover talents, aiming at the frontier of medical scientific development (1). To achieve this objective, diversified forms of education systems have been set for organizing undergraduate research including the undergraduate tutor system (2).

Originated from Oxford University in the fourteenth century, undergraduate tutor system has been continuously professionalized, institutionalized, and diversified for more than 600 years (3, 4). At present, it is a product of the combination of the British mentoring tradition and the American credit system and is also emulated and promoted by higher education in universal countries (5). The practice of undergraduate tutor system in Europe and the United States achieves the goals of teaching students according to their abilities, emphasizing on individual guidance, stimulating their interests, unlocking their potential, and cultivating their research awareness and abilities at an early stage (6). Through this peer-to-peer education mode of tutors and students, students can be nurtured by teachers in knowledge, governance, life, and character (7).

In Chinese undergraduate medical education, the undergraduate tutor system is emphasized more on early research training for students, which is consistent with the insistence of the Ministry of Education of China on the cultivation of students' innovative thinking and innovative ability to meet the national strategy. The Chinese government has gradually carried out educational reform to promote the development and cultivation of scientific research capabilities of medical undergraduates. There is no doubt that the emergence of the undergraduate tutorial system has found a breakthrough for students in need of scientific research. Tutors can provide students with guidance on scientific research ideas and design and help students to implement their plans. In addition, the purpose of the tutor system also includes shaping the personality of the students through the daily communication between the tutors and the students, so that the students can form correct values when they practice medicine in the future. Generally, different from graduate tutors who mostly focus on the scientific research ability, undergraduate tutors also concentrate on cultivating students' comprehensive quality, giving all-round guidance to undergraduates through lectures, clinical skills trainings, scientific research group meetings, and public welfare activities to promote students' physical and mental health and scientific research skills.

As for the 8-year medical students with less time to earn a doctorate degree, the undergraduate tutor system contains a greater significance since these students are demanded to obtain adept research skills and advanced innovating thinking when entering the doctor period (8). Therefore, these students have been chosen as the recipients of the undergraduate tutor system in several famous medical schools, including Xiangya Medical School of Central South University. Actually, the modality of the undergraduate tutor system highly impacts its effectiveness in the cultivation of the students. Many scholars have discussed its implementation, actual effect, and the possible influencing factors behind; however, no studies have used a controlled approach to specifically analyze the impact on scientific capability training at present (9–11). Therefore, we used the questionnaires to collect information from 8-year medical students who received two kinds of undergraduate tutor system and 5-year medical students who did not receive for control. As 8-year medical students received their medical education, 1 year later, than 5-year medical students, this study was conducted with 8-year medical students in grade 2016 and 5-year medical students in grade 2017. Analyzing the data, we ensured the detailed impact of different undergraduate tutor systems on the students' scientific research ability and related factors.

## Methods

### Ethical Approval

This study was approved by the Third Xiangya Hospital Ethics Committee. All participators signed an informed consent document as required by the institutional ethics committee.

### Survey Methods and Specific Questionnaire Design

Designed for a cross-sectional study, the questionnaire comprised three sections, namely, basic information, the subjective assessment of the undergraduate tutor system, and the scientific research ability and academic results.

The first block collected the characteristic data including gender, enrollment year and major, whether he/she was the only child in his/her family, the undergraduate tutor system style, the tutor's profession, whether his/her tutor was master's or doctoral supervisor, the average frequency of communication with the tutor, the guidance method of the tutor.

The second block reflected the subjective influence of the undergraduate tutor system including the view on scientific research, the daily mood, and vocational development planning *via* the 5-point Likert scale. The view on scientific research showed the impact on student's interest and attitude toward research. The daily mood was essential in assessing the participators' psychological health. Vocational development planning could reflect whether the tutors helped the students build professional awareness in a particular area. The view and mood were graded as follows: 1, completely negative; 2, kind of negative; 3, no change; 4, kind of positive; and 5, extremely positive; and the planning was graded as follows: 1 and 2, less clear; 3, no change; and 4 and 5, clearer. Besides, one single-choice question and one open-ended question were designed for exploring the greatest impact of the undergraduate tutor system on students in terms of research planning. These questions offered a more holistic perspective for us to comprehensively analyze the impact of the undergraduate tutor system on students except for simple academic indicators.

The third block estimated the academic impact results of the undergraduate tutor system. The interviewees were required to evaluate their familiarity with research skill projects including literature processing (i.e., retrieval, reading, and report), experimental operation (cell, molecular, immunological, and animal experiment), statistical analysis (i.e., software application and figure description), manuscript producing (i.e., basic medical experiment and clinical research results processing, writing skills, and illustration presentation) and innovative project design based on their assessment of their perceived competency using the 5-point Likert scale. The projects were graded as follows: 1, completely unskilled; 2, kind of unskilled; 3, normal; 4, kind of skilled; and 5, extremely skilled. The rating for each major item was calculated by averaging the ratings of the included minor items. In the evaluation of academic results, the students provided the number of the articles, which had been published including Chinese papers, patents, and Science Citation Index (SCI) papers. We also collected the types of their most published articles. The number of the students who presided or participated in the College Students' Innovative Entrepreneurial Training Plan Program was also counted.

### Questionnaire Distribution and Recovery

This was the first study of the effect and influence of the undergraduate tutor system on the undergraduate stage of Chinese 8-year medical program students in scientific research. All participants came from the 8-year medical program or the 5-year medical program in a Chinese well-known medical college, Xiangya Medical School of Central South University. The enrollment year of the participants was limited to 2016 for the 8-year medical program and 2017 for the 5-year medical program in this study. The students of 2016 in 8-year medical program could be considered no different from those of 2017 in 5-year medical program due to the same duration of the undergraduate stage. Before the initiation of the undergraduate tutor system, the students in 8-year medical program and 5-year medical program neither had any systematic scientific research training. All questionnaires were distributed and recovered anonymously in September 2021 with informed consents. All questionnaires were issued through an online platform named Wenjuanxing *via* convenience sampling.

### Questionnaire Reliability Analysis

The content of the questionnaire was reviewed by an expert panel including a medical education specialist, a student affairs management officer responsible for the undergraduate tutor system, an associate professor of health statistics, and several undergraduate tutors. The questionnaire was also presurveyed with 30 students and revised according to the feedback including adding an open-ended question. Cronbach's α of the total scale in this research (0.788) was examined for testing the internal consistency reliability.

### Questionnaire Data Grouping

The students in 8-year medical program had their own choices in choosing one kind of undergraduate tutor systems. The students in 5-year medical program had no distributed tutor. Students from different grades and programs were finally divided into three groups according to the different styles of undergraduate tutor systems they were receiving. In group A, one student had a single tutor for guidance. The students in group B had multiple tutors for each one. The students in group C had no personal tutor.

### Statistical Analysis of Questionnaire Data

The distribution of data was analyzed by the Kolmogorov-Smirnov test. The data with a skewed distribution were presented as the medians and interquartile range. The non-parametric test was applied for the data following a skewed distribution including the scores of students' research ability and the numbers of academic achievements. The chi-square test was used for comparing the constituent ratio of the data basic characteristics and the greatest subjective effect regarded by group A and B.

The ordinal logistic regression analysis was used to estimate the student's view on scientific research and the daily mood after the guidance of the tutor, for the scale of the multiple category outcome was not nominal but ordinal, and the rank ordering of the outcomes must be taken into account. We chose the continuation ratio model in the two analyses for our outcome variables were intrinsic five category variables, instead of variables that being subsequently grouped from originally continuous response variables (12). The ordinal logistic regression required the evaluation of the parallel lines assumption, which assumed that the values of the regression coefficient were equal in each category of the dependent variable. If the observations in each category of the dependent variable were heterogeneous or when the parallel lines assumption was rejected, ordinal logistic model could be used. The goal of the modeling was to explore tutor system-related factors associated with progressively positive attitude toward scientific research and the daily mood. In ordinal logistic regression, we selected “the type of tutorial system,” “the highest professional title of tutors,” “the frequency of guidance,” and “the way of guidance” as independent variables. The dependent variables or outcome variables were “the view on scientific research” and “the daily mood.” Multinomial logistic regression was used to estimate the student's vocational development planning after the guidance of the tutor, and the outcome variable has three levels, namely, “less clear,” “no change,” and “clearer,” with independent variables being the same as ordinal logistic regression. SPSS 22.0 was used for data analyses, with *P* < 0.05 as the level of statistical significance.

## Results

### Questionnaire Distribution and General Information of Groups

One week following the dissemination of 200 questionnaires, 194 (97.0%) of them were collected with complete answers, including 64 (33.0%) from a single tutor for the multiple students' system (group A), 72 (37.1%) from multiple tutors for the multiple students' system (group B), and 58 (29.9%) from the no tutor system for comparison (group C). Among them, 91 (46.9%) were male and 103 (53.1%) were female. Among the tutors, 124 (68.9%) of them came from the affiliated hospitals of Central South University, 53 (29.4%) of them came from School of Basic Medicine, and 3 (1.7%) of them came from School of Public Health, which showed that the students preferred to choose clinicians as their tutors. The most students' tutors (63.5%) were doctoral supervisors. As for the communication frequency, the most tutors (50.6%) mentored their students less than once a week. Although 54 (34.6%) students could receive the personal guidance from their tutors, the others were only instructed by the elder students (28.2%) or someone else with the tutors' regular care (17.3%). There was no statistical difference in the composition ratio of basic characteristics between groups. The whole basic characteristics of the participants are presented in Table 1.

**Table 1:** Demographic characteristics and basic information of the involved students and their tutors.

**Basic characteristics**	**Group A** **(*n =* 64) (%)**	**Group B** **(*n =* 72) (%)**	**Group C** **(*n =* 58) (%)**	***P***
**Gender**				
Male	30 (46.9%)	34 (47.2%)	27 (46.6%)	0.997
Female	34 (53.1%)	38 (52.8%)	31 (53.4%)	
**The only child or not**				
Yes	39 (60.9%)	49 (68.1%)	36 (62.1%)	0.648
No	25 (39.1%)	23 (31.9%)	22 (37.9%)	
**Tutors' source**				
Affiliated hospitals of Central South University	47 (74.6%)	60 (63.2%)	–	0.321
School of Basic Medicine	15 (23.8%)	33 (34.7%)	–	
School of Public Health	1 (1.6%)	2 (2.1%)	–	
**Tutors' level**				
Master's supervisor	16 (25.0%)	23 (31.9%)	–	0.670
Doctoral supervisor	43 (67.2%)	44 (61.1%)	–	
None	5 (7.8%)	5 (6.9%)	–	
**Average communication frequency**				
More than once a week	11 (17.2%)	8 (11.1%)	–	0.167
Once a week	11 (17.2%)	13 (18.1%)	–	
Less than once a week	29 (45.3%)	44 (61.1%)	–	
Basically none	13 (20.3%)	7 (9.7%)	–	
**Tutors' guidance method**				
Personally guide	21 (32.8%)	26 (36.1%)	–	0.080
Send other teachers to guide	2 (3.1%)	8 (11.1%)	–	
Send elder students to guide	14 (21.9%)	22 (30.6%)	–	
Someone else to guide with regular care	16 (25.0%)	10 (13.9%)	–	
No effective method	11 (17.2%)	6 (8.3%)	–	

*The chi-square test was applied for comparing the basic characteristics of the groups*.

### Subjective Influence of Different Tutor Systems

To investigate how the system affected the several essential individual views of students and their daily feeling, several indexes were used for quantification excluding group C who was not involved in the tutor system, and ordinal and multinomial logistic regression analyses were performed, and their variable assignment was listed in Table 2. The independent variable “the type of tutorial system” was divided into “single tutor for multiple students” and “multiple tutors for multiple students,” which was also the classification standard of group A and group B. The independent variable “the highest professional title of tutor(s)” was classified according to the title of the tutor by mixing group A and group B together. Similarly, taking group A and group B as a whole, the other two independent variables, namely, “the frequency of guidance” and “the way of guidance,” were set in line with their respective standards. In the study, the dependent variables “view on scientific research after the guidance of the tutor” and “daily mood after the guidance of the tutor” were ordinal categorical variables of five categories. Another outcome variable “vocational development planning after the guidance of the tutor” was a categorical variable of three categories.

**Table 2:** Variable assignment for ordinal and multinomial-logistic regression analysis of influential factors of different subjective influence of different tutor systems.

**Variables**	**Assignment**
**Outcome variables**	
View on scientific research after the guidance of the tutor	Completely negative = 1
	Kind of negative = 2
	No change = 3
	Kind of positive = 4
	Extremely positive = 5
Daily mood after the guidance of the tutor	Completely negative = 1
	Kind of negative = 2
	No change = 3
	Kind of positive = 4
	Extremely positive = 5
Vocational development planning after the guidance of the tutor	Completely unclear = 1
	Kind of unclear = 2
	No change = 3
	Kind of clear = 4
	Extremely clear = 5
**Independent variables**	
The type of tutorial system	Single tutor for multiple students = 1
	Multiple tutors for multiple students = 2
The highest professional title of tutor(s)	Master supervisor = 1
	Doctoral supervisor = 2
	No title = 3
The frequency of guidance	More than once a week = 1
	Once a week = 2
	Less than once a week = 3
	Hardly = 4
The way of guidance	Tutor's personal guidance = 1
	Assigning tutors from other teams = 2
	Assigning elder students of the tutor for guidance = 3
	Assigning others to guide but supervising periodically = 4
	No effective method = 5

*Group A (n1 = 64) and group B (n2 = 72) were included*.

The result of the ordinal logistic regression showed that the type of tutorial system, the frequency of guidance, and the way of guidance were significantly related to the view of students on scientific research (Table 3). In this section, the odds ratio (OR) value represented the ratio of the probability that a subcategory of the independent variable under consideration led to a higher rank of the ordinal 5 category outcome variable to the probability that the reference in the independent variable under consideration produced a higher rank of the ordinal dependent variable. In short, an OR > 1 indicated that the subcategory group was likely to hold a positive attitude. Students with only a single tutor were 0.477 times less likely to have more positive views on scientific research than those students with a supervisor group consist of multiple tutors [OR = 0.477, 95% confidence interval (CI) 0.244–0.934, *P* = 0.031]. In other words, group A students tended to have more negative view on scientific research than group B students. Interestingly, the OR (OR = 0.264, 95% CI 0.073–0.959, *P* = 0.043) indicated that those who received instruction less than one time a week were 0.264 times less likely to show greater optimism about scientific research as compared with students hardly receiving guidance. Compared with students who acquired guidance in no effective method, those mentored personally by tutor had a 5.397 times higher chance to be involved in more positive views on scientific research (OR = 5.397, 95% CI 1.232–23.644, *P* = 0.025) and others mentored by elder students were 4.959 times more likely to have more positive attitudes to scientific research (OR = 4.959, 95% CI 1.157–21.245, *P* = 0.031).

**Table 3:** Multiple ordinal logistic regression analysis of influential factors of view on scientific research after the guidance of the tutor.

**Explaining variables**	**Estimate**	***P*** **-value**	**Std. Error**	**Wald**	**OR**	**OR 95% CI**
**The type of tutorial system**						
Single tutor for multiple students = 1	−0.739	0.031*	0.342	4.661	0.477	0.244–0.934
Multiple tutors for multiple students = 2	0^a^					
**The highest professional title of tutor(s)**						
Master supervisor = 1	−1.047	0.203	0.822	1.620	0.351	0.07–1.759
Doctoral supervisor = 2	−0.849	0.277	0.781	1.181	0.428	0.093–1.978
No title = 3	0^a^					
**The frequency of guidance**						
More than once a week = 1	−0.175	0.823	0.783	0.050	0.840	0.181–3.895
Once a week = 2	0.131	0.860	0.747	0.031	1.140	0.264–4.927
Less than once a week = 3	−1.331	0.043*	0.658	4.094	0.264	0.073–0.959
Hardly = 4	0^a^					
**The way of guidance**						
Tutor's personal guidance = 1	1.686	0.025*	0.754	5.004	5.397	1.232–23.644
Assigning tutors from other teams = 2	0.950	0.281	0.881	1.161	2.585	0.459–14.549
Assigning elder students of the tutor for guidance = 3	1.601	0.031*	0.742	4.652	4.959	1.157–21.245
Assigning others to guide but supervising periodically = 4	1.421	0.074	0.795	3.197	4.139	0.872–19.645
No effective method = 5	0^a^					
**Threshold**						
**View on scientific research**						
Completely negative = 1	−4.105		0.785	27.348		
Kind of negative = 2	−2.547		0.681	13.999		
No change = 3	−0.677		0.645	1.102		
Kind of positive = 4	1.567		0.662	5.602		

*View on scientific research after the guidance of the tutor: 1 = Completely negative, 2 = kind of negative, 3 = no change, 4 = kind of positive, and 5 = extremely positive*.

Test of parallel lines: likelihood ratio χ2 (30) = 32.23, P = 0.357; model fitting information: likelihood ratio χ2 (10) = 25.72, P = 0.004, pseudo R^2^ = 0.184. P < 0.05 was indicated in bold. ^*^P < 0.05. Group A (n1 = 64) and group B (n2 = 72) were included.

*0^a^: This parameter was set zero because it was redundant. OR: odds ratio; CI: confidence interval*.

As for daily mood, it was influenced by the way of guidance according to findings of ordinal logistic regression (Table 4). Taking the way of no effective method for reference, the three methods, namely, tutor's personal guidance (OR = 8.513, 95% CI 1.890–38.344, *P* = 0.005), guidance from elder students of the tutor (OR = 4.856, 95% CI 1.122–21.011, *P* = 0.034), and guidance from others but supervised periodically by the tutor (OR = 4.967, 95% CI 1.027–24.022, *P* = 0.046), were 8.513, 4.856, and 4.967 times more likely to improve students' daily mood by at least one level, respectively.

**Table 4:** Multiple ordinal logistic regression analysis of influential factors of daily mood after the guidance of the tutor.

**Explaining variables**	**Estimate**	***P*** **-value**	**Std. Error**	**Wald**	**OR**	**OR 95% CI**
**The type of tutorial system**						
Single tutor for multiple students = 1	−0.456	0.177	0.338	1.821	0.634	0.327–1.229
Multiple tutors for multiple students = 2	0^a^					
**The highest professional title of tutor(s)**						
Master supervisor = 1	−0.965	0.243	0.826	1.365	0.381	0.075–1.923
Doctoral supervisor = 2	−1.165	0.139	0.788	2.185	0.312	0.067–1.462
No title = 3	0^a^					
**The frequency of guidance**						
More than once a week = 1	0.010	0.989	0.778	0.000	1.010	0.220–4.641
Once a week = 2	−0.078	0.916	0.740	0.011	0.925	0.217–3.940
Less than once a week = 3	−1.112	0.088	0.652	2.907	0.329	0.092–1.181
Hardly = 4	0^a^					
**The way of guidance**						
Tutor's personal guidance = 1	2.142	0.005**	0.768	7.778	8.513	1.890–38.344
Assigning tutors from other teams = 2	1.046	0.239	0.888	1.387	2.845	0.499–16.210
Assigning elder students of the tutor for guidance = 3	1.580	0.034*	0.747	4.471	4.856	1.122–21.011
Assigning others to guide but supervising periodically = 4	1.603	0.046*	0.804	3.974	4.967	1.027–24.022
No effective method = 5	0^a^					
**Threshold**						
**Daily mood**						
Completely negative = 1	−4.099		0.812	25.478		
Kind of negative = 2	−2.419		0.678	12.744		
No change = 3	−0.302		0.642	0.222		
Kind of positive = 4	1.550		0.658	5.548		

*Daily mood after the guidance of the tutor: 1 = Completely negative, 2 = kind of negative, 3 = no change, 4 = kind of positive, and 5 = extremely positive*.

Test of parallel lines: likelihood ratio χ2 (30) = 43.62, P = 0.052; model fitting information: likelihood ratio χ2 (10) = 23.29, P = 0.010, pseudo R^2^ = 0.168. P < 0.05 was indicated in bold. ^*^P < 0.05, ^**^ P < 0.01. Group A (n1 = 64) and group B (n2 = 72) were included. 0^a^: This parameter was set zero because it was redundant. OR: odds ratio; CI: confidence interval.

When investigating the vocational development planning of students after the guidance of the tutor by performing the multinomial logistic regression analysis, we found that the highest professional title of tutors had significant influence on this dependent variable (Table 5). Surprisingly, compared with students whose tutors without professional titles, students with tutors of master supervisors (OR = 28.505, 95% CI 1.504–540.37, *P* = 0.026) were 28.505 times more likely to be unclear about their future career development and scientific research planning. Moreover, it was gratifying that more than half of the students in both “single tutor for multiple students” and “multiple tutors for multiple students” were clearer about their vocational development plans although not statistically significant.

**Table 5:** Multinomial logistic regression analysis of influential factors of vocational development planning after the guidance of the tutor.

	**Vocational development planning**					**OR (95% CI)**	
**Factors**	**Clearer *n* (%)**	**No change *n* (%)**	**Less clear *n* (%)**	**χ^2^**	**P**	**Clearer**	**Less clear**
**The type of tutorial system**							
Single tutor for multiple students	34 (53.1%)	13 (20.3%)	17 (26.6%)	2.175	0.337	1.292 (0.517–3.231)	1.764 (0.566–5.493)
Multiple tutors for multiple students	38 (52.8%)	21 (29.2%)	13 (18.1%)			Reference category	
**The highest professional title of tutor (s)**							
Master supervisor	19 (48.7%)	8 (20.5%)	12 (30.8%)	3.697	0.449	0.438 (0.030–6.455)	**28.505 (1.504**–**540.37)**
Doctoral supervisor	48 (55.2%)	22 (25.3%)	17 (19.5%)			0.403 (0.031–5.243)	10.045 (0.616–163.67)
No title	5 (50.0%)	4 (40.0%)	1 (10.0%)			Reference category	
**The frequency of guidance**							
More than once a week	14 (73.7%)	3 (15.8%)	2 (10.5%)	20.844	0.002	1.192 (0.109–13.019)	0.120 (0.007–1.947)
Once a week	20 (83.3%)	4 (16.7%)	0 (0.0%)			1.393 (0.144–13.429)	–
Less than once a week	31 (42.5%)	22 (30.1%)	20 (27.4%)			0.421 (0.057–3.098)	0.235 (0.030–1.832)
Hardly	7 (35.0%)	5 (25.0%)	8 (40.0%)			Reference category	
**The way of guidance**							
Tutor's personal guidance	28 (59.6%)	11 (23.4%)	8 (17.0%)	9.416	0.308	8.039 (0.580–111.34)	0.904 (0.104–7.856)
Assigning tutors from other teams	4 (40.0%)	4 (40.0%)	2 (20.0%)			4.825 (0.281–82.974)	0.581 (0.047–7.242)
Assigning elder students of the tutor for guidance	21 (58.3%)	7 (19.4%)	8 (22.2%)			11.24 (0.820–154.13)	1.603 (0.188–13.67)
Assigning others to guide but supervising periodically	15 (57.7%)	6 (23.1%)	5 (19.2%)			10.60 (0.689–163.11)	0.898 (0.090–8.981)
No effective method	4 (23.5%)	6 (35.3%)	7 (41.2%)			Reference category	

*Group A (n1 = 64) and group B (n2 = 72) were included*.

*OR (odds ratio), the probability of students likely to change clarity about their vocational development planning, compared with the reference. CI, confidence interval*.

**Figure 1:**
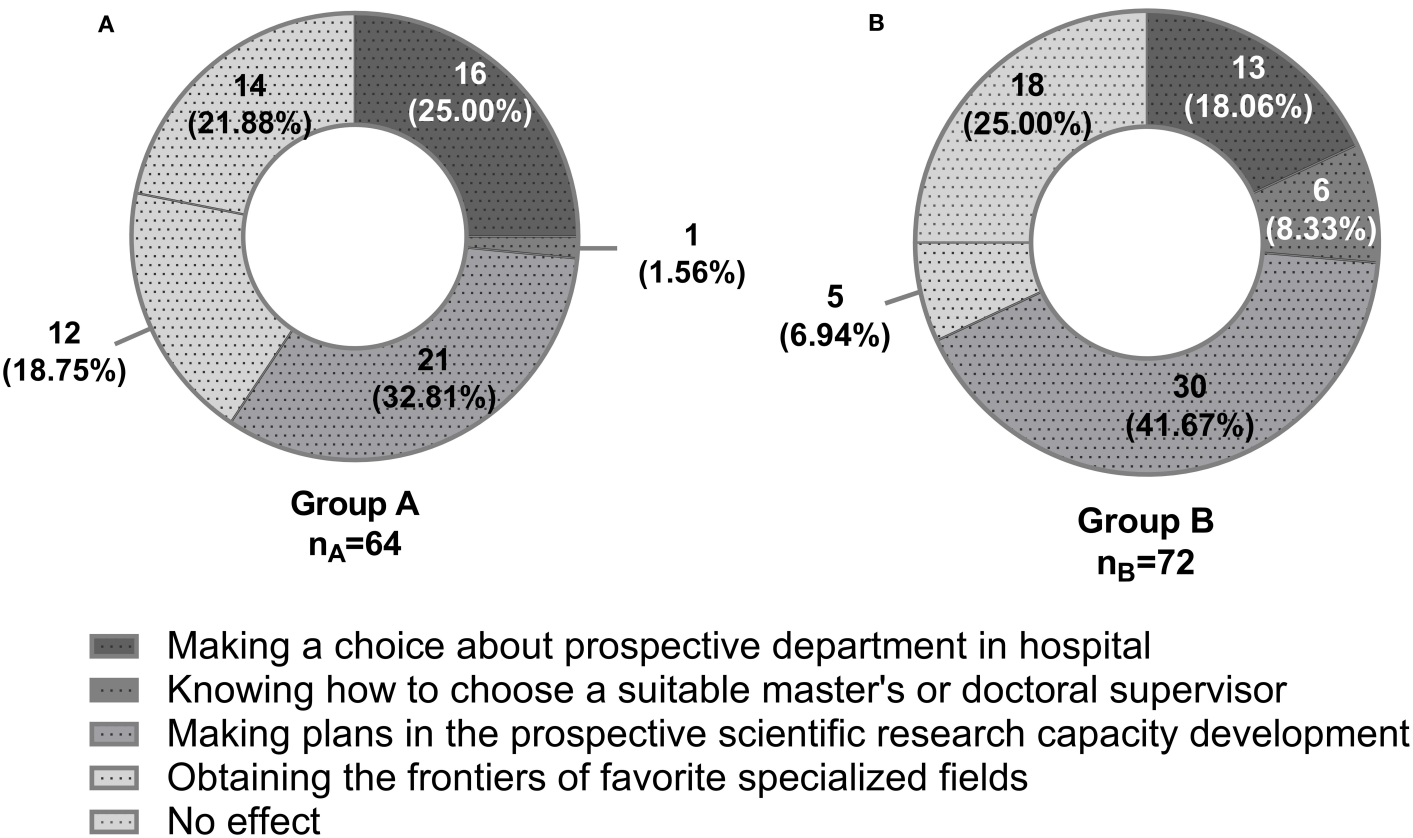
The greatest effect of the undergraduate tutor system on students. **(A)** Group A, single tutor for multiple students' system. **(B)** Group B, multiple tutors for multiple students' system. The chi-square test was applied for the comparison.

In both groups, most students (i.e., 32.8% and 41.7%) considered that the greatest effect of the undergraduate tutor system on students was helping them make their plans in the prospective scientific research capacity development (Figure 1). In group A, the students also deemed that the single tutor for multiple students' system assisted them most with making a choice about prospective department in hospital (25.0%) or obtaining the frontiers of favorite specialized fields (18.8%). The students in group B believed that the multiple tutors for multiple students' system gave them the greatest support in making a choice about prospective department in hospital (18.1%) or knowing how to choose a suitable master's or doctoral supervisor (8.3%). However, there were still 21.9% students of group A and 25.0% students of group B, though the undergraduate tutor system was not very effective. The constituent ratio of the two groups on the greatest subjective effect remained different with statistical significance (*P* < 0.001).

In the open-ended question, the most mentioned answer was about starting the undergraduate tutor system earlier and providing more detailed information introducing the tutors. Most students held a positive attitude toward their tutors; however, some appealed to the school for a stricter tutorial selection threshold. They considered that the tutors should be the ones with sufficient time in guiding students instead of busy academic leaders.

### Scientific Research Ability and Academic Results of Different Tutor Systems

**Figure 2:**
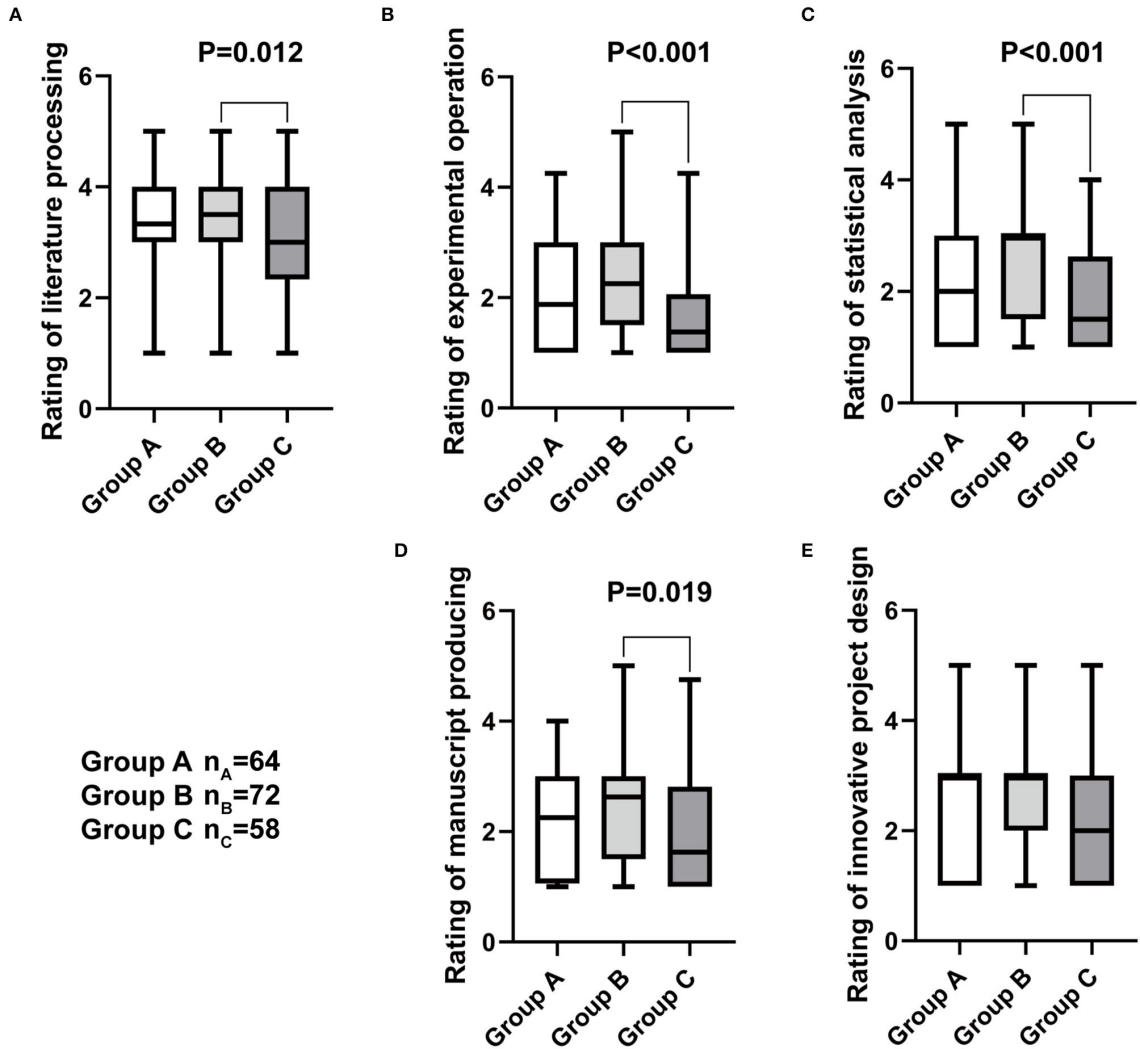
The box plots of the students' research ability rating with the 5-point Likert scale. **(A)** The average rating of literature processing. **(B)** The average rating of experimental operation. **(C)** The average rating of statistical analysis. **(D)** The average rating of manuscript producing. **(E)** The average rating of innovative project design. The non-parametric test was applied for the comparison.

In terms of the assessment of the students' scientific research ability, five projects were employed to score them with the 5-point Likert scale (Table 6). In the literature processing (i.e., retrieval, reading, and report), the group B obtained 3.50 (1.00) points, apparently higher than the group C who only got 3.00 (1.67) points (*P* = 0.012, Figure 2A). The group B also had the significantly higher rating of 2.25 (1.50) points in experimental operation (i.e., cell, molecular, immunological, and animal experiment) comparing with the group C (*P* < 0.001, Figure 2B). Students in the group B earned 3.00 (1.50) points in the statistical analysis (i.e., software application and figure description), showing a significant difference between 1.50 (1.63) points acquired by the group C (*P* < 0.001, Figure 2C). With regard to the manuscript producing including basic medical experiment and clinical research results processing, writing skills, and illustration presentation, the group B had 2.63 (1.50) points, significantly higher than the group C (*P* = 0.019, Figure 2D). All the three groups displayed no statistical significance in the comparison of the innovative project design (*P*>0.05, Figure 2E). In addition, the two groups of the undergraduate tutor system exhibited no significant different in all the examined programs (*P*>0.05, Figure 2).

**Table 6:** The assessment of students' scientific research ability in literature processing, experimental operation, statistical analysis, manuscript producing, and innovative project design with the 5-point Likert scale.

**Scientific research ability**	**Median**	**Inter-quartile range**	***P*** **-value compared with Group C**
**Literature processing**			
Group A	3.33	1.00	0.090
Group B	3.50	1.00	0.012*
Group C	3.00	1.67	–
**Experimental operation**			
Group A	1.88	2.00	0.119
Group B	2.25	1.50	< 0.001***
Group C	1.38	1.06	–
**Statistical analysis**			
Group A	2.00	2.00	0.196
Group B	3.00	1.50	<0.001***
Group C	1.50	1.63	–
**Manuscript producing**			
Group A	2.25	1.94	0.221
Group B	2.63	1.50	0.019*
Group C	1.63	1.81	–
**Innovative project design**			
Group A	3.00	2.00	0.517
Group B	3.00	1.00	0.087
Group C	2.00	2.00	-

The non-parametric test was applied for the comparison. P < 0.05 was indicated in bold. ^*^P < 0.05, ^***^P < 0.001. Group A, n1 = 64; group B, n2 = 72; and group C, n3 = 58.

**Figure 3:**
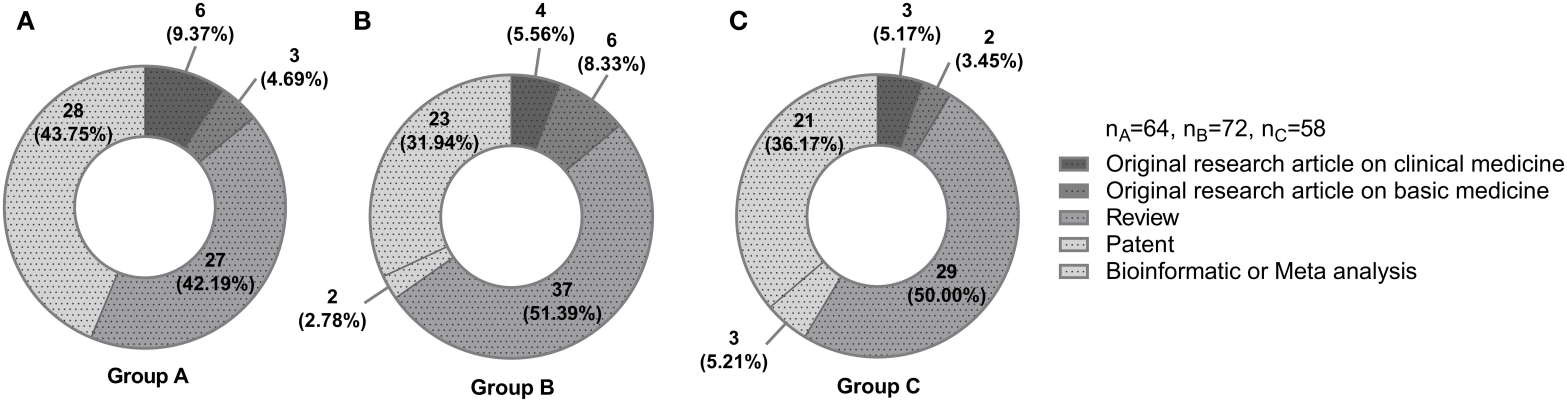
The most published types of articles by students. **(A)** Group A, single tutor for multiple students' system. **(B)** Group B, multiple tutors for multiple students' system. **(C)** Group C, no tutor system.

To estimate the academic results of different groups, we collected the information about the students' published papers and other associated data. Totally, the students participating in the undergraduate tutor system delivered 44 Chinese papers, and had 27 patents granted. They also published 87 SCI papers, 64 of which were first, second, or corresponding authors, accumulating the impact factor of 320. They hosted 96 College Students' Innovative Entrepreneurial Training Plan Programs and took part in 294 programs. Compared with the group C, the group A and B had significantly more SCI papers published by students as first, second, or corresponding authors (*P* = 0.029, *P* = 0.047). These two groups also joined in apparently more National college students' innovation and entrepreneurship training programs with statistical significance (*P* = 0.003, *P* < 0.001). The most published types of articles by students in the group A were bioinformatics or meta-analysis (43.8%) and reviews (42.2%) (Figure 3A). Both the group B and C preferred to deliver reviews (51.4% and 50.0%) (Figures 3B,C).

## Discussion

The undergraduate tutor system is one of the most distinctive parts of China's 8-year medical education (13). The government and schools hope to allocate appropriate tutors for students to lead them in scientific research and clinical skills learning. In this research of the undergraduate tutor system, we found that different systems affected students in several aspects except for scientific research. The results showed that students were more inclined to choose doctors from hospitals as their tutors, most of whom were master and doctoral supervisors. However, due to their heavy tasks in administration, teaching, research, and clinic, the frequency of guidance was insufficient, resulting in difficulty for providing timely and effective guidance to help students solve their problems (14). As Russell et al. reported, the most common suggestions that students made about how to improve undergraduate research programs was increased or more effective faculty guidance (15). Many students were not supervised by tutors themselves, indicating the fact that the undergraduate tutor system might not achieve the desired aims. Benefiting from the undergraduate tutor system, nonetheless, they might be mentored by postgraduates, technical assistants, and other students (16). It may be more effective to choose young teachers without master's or doctoral supervisor title soon after graduation of doctor as tutors, who are more leisure to personally guide students and most likely to combine enthusiasm with interpersonal, organizational, and research skills resulting in facilitating positive outcomes (17).

There was basically no statistical difference between the types of the undergraduate tutor system (i.e., single tutor for multiple students or multiple tutors for multiple students) in students' subjective assessment, except for views on scientific research, which might be caused by the fact that the students were more likely to bring up their interest in scientific research under the co-cultivation of multiple tutors than a single tutor. The students' subjective assessment was also greatly affected by other factors related to the undergraduate tutor system. Previous studies showed that the scientific research training guided by tutors could make students feel confident in performing tasks in their future career (18, 19). It was interesting that the influence of the tutors who instructed students less than one time a week was even worse than that of hardly tutoring students. Students responded that in this case, the tutors would arrange a large number of tasks at once by students themselves to process and check back several weeks later, which seriously struck the students' attitude toward scientific research. When it came to the way of guidance, we found that the students guided by the tutors personally or by the senior students were more active in scientific research and daily mood, indicating that the tutors must strengthen the connection with their students and truly care for their students. However, if the tutor arranged other teachers to guide the students, there was no statistical difference in the subjective assessment of these students compared with those who hardly got guidance. The reason might be that these temporarily arranged teachers were difficult to enter the heart of the students, conduct in-depth conversation, and establish a good relationship. Similarly, Pan et al. also discovered that the toughest difficulties for students in the cultivation of scientific research and innovation ability were insufficient communication frequency with their tutors and a lack of guidance at important stages hindering the establishment of scientific research thinking (20). The students whose tutors without professional titles were clearer about their career planning compared with the ones with tutors of master supervisors. Combined with the feedback of the open-ended question, young tutors who have just obtained a doctorate in medicine are more able to guide students in career planning for two possible reasons, namely, (1) they are newly experienced people who can summarize and share the experience suitable for the current situation and (2) as they just enter the work position, young doctors are more familiar with the current development status of the field than elder tutors. They also know better how to keep up with the time and communicate with students closer.

As the cross-sectional analytical study reported by Peng et al., the undergraduate research programs could help Chinese medical undergraduates build interest in scientific research and develop scientific thinking and basic research capacities (21). The undergraduate tutor system especially, the system of multiple tutors for multiple students also significantly improved the confidence and satisfaction of students in mastering various scientific research abilities. The students in group B belonging to this system had a higher literature processing level than group C without tutors, which meant that the undergraduate tutor system could help them obtain a wider range of academic theories and scientific research ideas, thereby laying a solid foundation for the formation of their own scientific research thought (22). Cell culture, molecular biological experiment, immunological experiment, and animal experiment are the basis of scientific research. Training in these items can greatly improve students' scientific research skills. Students in group B possessed multiple tutors from different specialized fields with different commonly used experimental skills. Therefore, they owned more opportunities to experience and learn more diverse experiments, which greatly improved their experimental ability. They also acquired better capability in statistical analysis, which reflected the students' enhanced scientific thinking and data processing ability. For paper writing, one of the most important abilities is logical thinking based on the analysis of experimental results. Students in group B had multiple tutors who could provide them with more projects for data processing and writing training, and more training would naturally help students master the skills and increase their confidence (20). Proposing and designing scientific research projects reflect students' sensitivity to scientific research, which is also a way to apply their theoretical knowledge, innovative thinking, and comprehensive quality in scientific research (23, 24). In the concrete implementation, students often explore together, inspire each other, and collaborate to accomplish the common goal (25). However, there was no significant difference between group A or B and group C in the design of innovative projects, which might be associated with short training time. Good ideas or thoughts are not only suddenly formed within 2 years but require years of accumulation in order to build scientific and rigorous innovative thinking (26). The lack of time seemed to be a major drawback of the 8-year undergraduate tutor system, which was also reflected in the open-ended question.

In terms of academic achievements, students with the undergraduate tutor system were not particularly better than the non-tutorial students, except for the number of college students' Innovative Entrepreneurial Training Plan Programs they participated in. This program is a national project in which the undergraduate students, under the guidance of their supervisors, independently complete innovative research or entrepreneurial project design, implementation, report writing, and academic achievement exchanges. More students of undergraduate tutor system participating in this program exhibited that the system could promote students' interest in scientific research, and they were more willing to know the operation of scientific research projects. As to why the system ultimately failed to produce enough academic achievements, we speculated that during the undergraduate period, the students had heavy learning tasks and insufficient time to participate in scientific research. In a multicenter cross-sectional questionnaire-based study, correspondingly, Li et al. pointed out that the 8-year program students generally felt conducting research was stressful and difficult, the greatest obstacle of which was a lack of time due to heavy workloads (27). Even if students are trained well within a limited time, more time is needed to produce scientific articles. Moreover, the 8-year program students are faced with the problem of selecting a doctoral supervisor, and the tutor in undergraduate stage is not necessarily the supervisor in the field of interest, which may lead to the switch of the research field. Also, some students think that the future will be more variable and their interest will change, who will not devote themselves to the output of papers but the cultivation of scientific research ability. As for the types of publications, most students with limited time and energy published reviews, bioinformatics, and meta-analysis, which need a shorter creation time, smaller cost, and faster publication. In the initial stage of scientific research, by reviewing the existing literature or utilizing databases in a certain field, students can understand the research progress of the problem to be studied, expand the research ideas and methods, and better master information retrieval and statistical knowledge. We believe that writing such articles is also beneficial to the cultivation of students' scientific research ability (28).

To sum up, we think that the undergraduate tutor system should be improved in the following directions: (1) The form of multiple tutors can be retained, but the tutor group should be young, and there is no need to pursue master or doctor tutors who are always in a heavy task. The focus is to ensure enough time for close contact with students. (2) The school should pay more attention to the influence of the undergraduate tutor system on students' daily life and future development. It can be considered to conduct training related to psychological counseling and career planning before the tutors take office. (3) It is not necessary to quantify the effect of the tutor system by specific academic achievement indicators such as the number of SCI papers published but to concern the training efficacy on students' ability. Frankly, our research also has some limitations needing enhancement in the following aspects: (1) The sample size is insufficient with only one school to investigate. Multicenter research can be supplemented in the future. (2) This research is a cross-sectional study, and a cohort or retrospective study may be considered in the future. (3) It may expand the implementation group of the undergraduate tutor system among 5-year medical students, which will make the impact of it more convincing.

## Conclusion

This was the first study to evaluate the diverse influence of the undergraduate tutor system on Chinese 8-year medical students in scientific research. We found some discrepancies between students with or without different types of systems, which was useful and helpful to better devise its implement way during the medical education reform. The subjective assessment of the undergraduate tutor system was related with certain factors such as communication frequency and guidance way. The students with multiple tutors for multiple students mastered higher level of scientific research ability including literature processing, experimental operation, statistical analysis, manuscript producing, and innovative project design. However, their academic publication showed no significant difference. The positive effect of the undergraduate tutor system should be affirmed; however, it still requires amelioration including selecting more young teachers with Ph.D. or M.D. as tutors for their more attention in psychological concern, daily care, and career planning guidance.

## Data Availability Statement

The original contributions presented in the study are included in the article/supplementary materials, further inquiries can be directed to the corresponding authors.

## Ethics Statement

The studies involving human participants were reviewed and approved by Third Xiangya Hospital Ethics Committee. The patients/participants provided their written informed consent to participate in this study.

## Author Contributions

The research idea was proposed by JW and PR. YL, HZ, FW, MZ, and JW took responsibility for manuscript writing and revising. All authors participated in data collecting and analyzing and contributed to the article and approved the submitted version.

## Funding

This study was supported by 2021 Innovation and Entrepreneurship Educational Reform Project of Central South University (No. 61, 62) and Educational Reform Project of Central South University (2020jy179).

## Conflict of Interest

The authors declare that the research was conducted in the absence of any commercial or financial relationships that could be construed as a potential conflict of interest.

## Publisher's Note

All claims expressed in this article are solely those of the authors and do not necessarily represent those of their affiliated organizations, or those of the publisher, the editors and the reviewers. Any product that may be evaluated in this article, or claim that may be made by its manufacturer, is not guaranteed or endorsed by the publisher.
